# Comparative Efficacy and Safety of Baricitinib Against Traditional Therapies in Severe Alopecia Areata: A Retrospective Cohort Study

**DOI:** 10.1111/jocd.16666

**Published:** 2024-11-20

**Authors:** Athanasios J. Stefanis, Tomas Dolezal, Spyridon Gkalpakiotis, Petr Arenberger

**Affiliations:** ^1^ Department of Dermatovenerology, Third Faculty of Medicine Charles University and University Hospital Kralovske Vinohrady Prague Czech Republic; ^2^ Value Outcomes Prague Czech Republic

**Keywords:** alopecia areata, baricitinib, baricitinib efficacy, baricitinib safety, JAK inhibitors

## Abstract

**Introduction:**

Alopecia areata is a common autoimmune disease which results in reversible hair loss. Janus kinase inhibitors are prescribed for severe alopecia areata with encouraging results. There are no studies comparing the efficacy and safety of Janus kinase inhibitors to traditional treatment options, such as topical immunomodulators and traditional immunosuppressants.

**Aims:**

To retrospectively compare the efficacy and safety of baricitinib, an approved Janus kinase inhibitor, to other treatments for severe AA during a 6‐month treatment period.

**Materials/Methods:**

We included patients with newly presenting, relapsing or treatment‐resistant alopecia areata with Severity of Alopecia Tool (SALT) score ≥ 50, for the period between July 2021 and July 2023. Medical histories were reviewed and possible side effects were recorded. Primary endpoints were SALT ≤ 20 and SALT ≤ 10 after 6 months of treatment.

**Results:**

Seventy‐five patients (53 females) were divided into three groups: topical immunomodulators (51 patients); baricitinib (19 patients); and a group receiving pulsed intramuscular corticosteroids or traditional immunosuppressants (11 patients). Twenty‐one patients received more than one treatment options within 2 years. After 6 months, the baricitinib group showed superior efficacy with 32% and 26% of patients achieving SALT ≤ 20 and SALT ≤ 10, compared to 12% and 9% in both other groups. Baricitinib demonstrated better secondary outcomes (50% and 90% reduction from initial SALT scores). All treatments exhibited mild‐to‐moderate and expected side effects. Weight gain, which had not been reported in clinical trials for alopecia areata, was observed in three baricitinib‐treated patients.

**Conclusion:**

Baricitinib was superior to traditional treatments for severe alopecia areata after 6 months. Weight gain concerned 16% of patients receiving baricitinib.

## 1 Introduction

Alopecia areata (AA) is a condition of autoimmune origin characterized by reversible hair loss [1]. It affects approximately 2% of the global population, regardless of ethnicity or gender [1]. The underlying mechanism involves the production of auto‐antibodies that attack the hair follicle cells, disrupting the normal hair growth cycle and resulting in hair loss [2]. The condition can manifest as patchy hair loss in various areas of the body, most commonly the scalp, but also including facial and body hair [3]. In some cases, it can progress to complete baldness on the scalp (alopecia totalis: AT) or even loss of all body hair (alopecia universalis: AU) [4]. While AA is typically described as a temporary condition, complete hair regrowth is observed in only about 65% of patients within 5 years, and relapses can occur over a period of up to 20 years after the initial episode [2]. AA has been found to have associations with other autoimmune disorders, such as atopic dermatitis, rhinitis, asthma, autoimmune thyroid diseases (AITD), vitiligo, pernicious anemia, and Type 1 diabetes [5].

The treatment of AA aims to suppress the autoimmune response and promote hair regrowth [6]. In cases with limited involvement, topical or intralesional corticosteroids are commonly utilized with varying results. For severe AA, where more than 50% of the scalp is affected, preferred options include topical immunomodulators like diphencyprone (DCP), contact sensitizers like anthralin, as well as systemic immunosuppressive drugs such as cyclosporine, methotrexate, or oral corticosteroids [6]. Despite the array of available medications, managing severe AA remains challenging, as traditional treatments often exhibit limited effectiveness and significant side effects.

The discovery of the JAK–STAT pathway and its relevance to AA pathogenesis has marked a new era in treating this autoimmune condition [2]. JAK inhibitors (JAKi) have shown promising and consistent results in addressing AA and represent the first class of drugs registered for the treatment of severe AA since May 2022 [2]. However, in many countries, they are not yet considered the primary option, and traditional treatments continue to be employed. Currently, no studies have directly compared the efficacy of JAKi to traditional treatment options for severe AA.

The objective of this study is to retrospectively assess and compare the efficacy and safety of various treatments, including JAKi, provided at our hospital for patients with severe AA for 6 months over a 2‐year period.

## 2 Methods

### 2.1 Study Design

This retrospective chart review took place at the outpatient department of a metropolitan hospital using patient records for the period between July 2021 and July 2023. Data were extracted from an AA‐specific registry which was developed in June 2021. All patients provided informed consent for the use of their data for research purposes upon their initial visit to our hospital. The study was approved by the institutional review board of our hospital.

### 2.2 Study Population

We assessed the medical records of our hospital and included all patients, adults and children with severe (SALT ≥ 50), newly presenting, relapsing or treatment‐resistant AA (no hair growth for 6 months) presenting to our outpatient department. SALT score is determined by adding the hair loss percentage in the various areas of the scalp. Patients already receiving treatment at the start of our study, with disease on remission during the study period, concomitant presence of other alopecias (androgenetic alopecia stage III and above in Norwood–Hamilton scale or Stage II and above according to Ludwig classification, telogen effluvium, trichotilomania, congenital alopecia, tinea capitis, syphilis of the scalp, scarring alopecias), and patients not consenting with data sharing were excluded. We also did not include records without documentations on treatment progress using SALT score and patients who were lost in follow‐up or opted to self‐withdraw from the proposed treatment before a 6‐month period.

### 2.3 Study Size

For statistical analysis of treatment efficacy in our retrospective chart review we included all treatment groups with at least 10 patients in order to present statistically significant and clinically useful results. We also collected safety data from all patients included in the study.

### 2.4 Clinical Assessments

Data were collected from participants during the clinical examination and from the hospital database. Age, gender, weight, height, personal history and family history, medicines taken, known allergies, severity, and clinical type of alopecia were recorded. The extent of alopecia and the efficacy of treatment were measured using the Severity of Alopecia Tool (SALT) [7]. The presence or absence of eyebrows, eyelashes, facial and body hair was also recorded. Safety assessments included all reported adverse events and abnormal clinical laboratory tests.

### 2.5 Efficacy Endpoints

The efficacy results after 6 months of treatment were assessed by measuring the percentage of patients who reached specific SALT scores, namely SALT score ≤ 20 (indicating ≤ 20% scalp hair loss) and SALT score ≤ 10 (indicating ≤ 10% scalp hair loss), as per findings from previous studies [8, 9]. Secondary outcome measures included the percentages of patients who achieved at least a 50% improvement (SALT50) and at least a 90% improvement (SALT90) from their baseline SALT scores.

### 2.6 Statistical Methods

Descriptive statistics were used to describe patient characteristics; frequencies and percentages were provided for categorical variables. Quantitative variables were represented as mean and standard deviation (SD) for continuous variables or median and interquartile range for non‐parametric continuous variables. Normality of numerical variables of baseline characteristics of all patients was tested using Kolmogorov–Smirnov test. The baseline characteristics of the three unequally sized treatment groups were compared using the Kruskal–Wallis test, for numerical data, and chi‐squared test for categorical data. Imputation techniques (individual mean substitution and last observation carried forward) were applied to missing data regarding treatment efficacy. The statistical significance level was set to 0.05. All statistical analyses were conducted using Microsoft Excel and IBM SPSS Statistics (Version 24.0; IBM Corp., Armonk, NY).

## 3 Results

We recorded and accessed 389 patients with AA who were in our clinic during the study period. Among them, 55 patients were already receiving treatment, 198 patients were diagnosed with SALT < 50, 44 patients terminated their treatment choice before 6 months (26 because of lack of efficacy and 18 because of intolerance), and 17 were lost to follow‐up. Based on our criteria, 75 patients with severe AA were considered eligible and included in our study.

### 3.1 Baseline Characteristics

The majority of our patients were females (70.7%), with an average age of 35 years and a mean SALT score of 87.49 (SD = 17.29), as presented in Table 1. The primary clinical type observed was AU, accounting for 45.3% of cases, followed by alopecia reticularis (26.7%), which is severe hair loss in a reticular pattern without distinct bald patches [10]. One out of two patients experienced hair loss on the face or body.

**TABLE 1:** Epidemiology and clinical features of patients with severe alopecia areata.

Variable	Total, *n* (%), *N* = 75
Age at presentation, years	
Mean ± SD	35.1 ± 16.8
Range (median)	3–70 (35)
Gender	
Females	53 (70.7)
BMI (kg/m^2^)	
Mean ± SD	23.4 ± 3.8
Clinical types	
Scalp	
Multilocularis	11 (14.7)
Reticularis	20 (26.7)
Ophiasis	1 (1.3)
Sisaipho	1 (1.3)
Diffusa	2 (2.7)
Totalis	6 (8.0)
Universalis	34 (45.3)
Other areas affected	
Barbae	38 (50.7)
Eyebrows	49 (65.3)
Eyelashes	41 (54.7)
Nails	36 (48.0)
Body hairs	37 (49.3)
Clinical severity, SALT	
Mean ± SD	87.5 ± 17.3
Range (median)	50–100 (100)
Age of onset, years	
Mean ± SD	25.6 ± 17.0
Range (median)	1–70 (25.0)
Current disease duration, months	
Mean ± SD	42.5 ± 65.2
Range (median)	1–300 (18)
No. of total hair loss, past	
Mean ± SD	0.5 ± 0.6
Range (median)	0–2 (0)
Personal history	
Atopic diseases	38 (50.7)
AD (present/past)	30 (40.0)
Asthma	8 (10.7)
Allergic rhinitis/conjunctivitis	19 (25.3)
AITD	21 (28.0)
Receiving thyroxine	14 (18.7)
After thyroidectomy	1 (1.3)
Receiving anti‐thyroid drugs	0
Vitiligo	5 (6.7)
Family history	
Atopic diseases	26 (34.7)
AD (present/past)	16 (21.3)
Asthma	10 (13.3)
Allergic rhinitis/conjunctivitis	7 (9.3)
AITD	10 (13.3)
Alopecia areata	9 (12.0)
Psoriasis	4 (5.3)
Rheumatoid arthritis	4 (5.3)
Vitiligo	2 (2.7)

Abbreviations: AD, atopic dermatitis; AITD, autoimmune thyroid disease; BMI, body mass index; *N*, *n*, number of patients; SALT, Severity of Alopecia Tool; SD, standard deviation.

More than 50% and 30% of patients reported a personal and family history of atopic diseases (atopic dermatitis, asthma, and allergic rhinitis/rhinoconjuctivitis), respectively (Table 1). Most common associated autoimmune diseases were AITD (28.0%) and vitiligo (6.7%).

The majority of subjects underwent treatment with topical immunomodulators, including DCP (*n*: 42) or a combination of DCP with anthralin (*n*: 9) (Table 2). The second largest cohort received baricitinib (*n*: 19), while 18 patients received other treatments, including intramuscular corticosteroid injections (*n*: 5), topical clobetasol (*n*: 4), systemic traditional immunosuppressants (*n*: 6), topical vitamin D (*n*: 1), anthalin (*n*: 1), and topical minoxidil (*n*: 1). Twenty‐one patients received more than one type of treatment over the 2‐year period. For data analysis, patients were divided into three treatment groups: topical immunomodulators with or without anthralin (topical IMMs); baricitinib; and a group incorporating intramuscular corticosteroid injections (CS) and traditional immunosuppresants (Trad. ISs).

**TABLE 2:** Treatments used for patients with severe alopecia areata in our study for at least 6 months.

Treatment	Route of administration	Dosage	Frequency
DCP: *N* = 42	Topical	0.001%–2%	Weekly
Baricitinib: *N* = 19	Oral	4 mg	Daily
DCP with anthralin: *N* = 9	Topical	0.5%–1%	Weekly + 5 times/week
Methylprednisolone acetate: *N* = 5	Intramuscular	40–80 mg	Monthly
Clobetasol: *N* = 4	Topical	0.5 mg/g	5–7 times/week
Methotrexate: *N* = 4	Oral	7.5–20 mg	Weekly
Cyclosporine: *N* = 1	Oral	5 mg/kg	Daily
Azathioprine: *N* = 1	Oral	2 mg/kg	Daily
Minoxidil: *N* = 1	Topical	5 mg/g	Daily
Vitamin D: *N* = 1	Topical	4.17 mcg/g	Daily
Anthralin: *N* = 1	Topical	1% g/g	Daily

Abbreviations: DCP, diphencyprone; *N*, number of patients.

### 3.2 Differences in Baseline Characteristics Among Treatment Groups

There were no significant variations in most baseline features (Table 3). Statistically significant variations emerged in clinical types, revealing a higher proportion of patients with alopecia reticularis in the topical IMMs group (31.4%; *p* = 0.041) in contrast to the baricitinib group where the majority presented with AU (73.7%; *p* = 0.035). A statistically significant difference was also observed in reported current disease duration. Patients in the baricitinib group had experienced hair loss for an average of 76.2 months compared to 24.6 months for those in the topical IMMs and 48.9 months for those receiving other treatments (CS+ Trad. ISs).

**TABLE 3:** Comparison of baseline characteristics in patients with severe alopecia areata treated with topical immunomodulators (IMMs), intramuscular corticosteroids (CS)/systemic traditional immunosuppressants (Trad. ISs) or baricitinib; SD, standard deviation.

Variable	Topical IMMs, *n* (%), *N* = 51	CS+ Trad. ISs, *n* (%), *N* = 11	Baricitinib, *n* (%), *N* = 19	*p*
Age at presentation, years				
Mean ± SD	34.6 ± 18.6	36.6 ± 17.5	40.3 ± 15.1	0.481
Range (median), years	10–70 (31)	13–59 (48)	20–70 (45)	
Gender (females)	37 (72.5)	8 (72.7)	11 (57.9)	0.479
BMI (kg/m^2^)				
Mean ± SD	23.7 ± 3.8	21.9 ± 1.0	23.4 ± 2.3	0.084
Clinical types, scalp				
Multilocularis	5 (9.8)	3 (27.3)	2 (10.5)	0.281
Reticularis	16 (31.4)	1 (9.1)	1 (5.3)	0.041*
Ophiasis	1 (2.0)	0	0	1
Sisaipho	1 (2.0)	0	1 (5.3)	0.606
Diffusa	1 (2.0)	0	0	1
Totalis	7 (13.7)	2 (18.2)	1 (5.3)	0.554
Universalis	20 (39.2)	5 (45.5)	14 (73.7)	0.035*
Clinical severity, SALT				
Mean ± SD	88.6 ± 16.9	87.4 ± 19.8	94.5 ± 11.7	0.265
Age of onset, years				
Mean ± SD	27.0 ± 19.5	28.0 ± 19.0	26.0 ± 16.0	0.958
Current disease duration, months				
Mean ± SD	24.6 ± 30.4	48.9 ± 48.5	76.2 ± 100.1	0.004**
No. of complete hair loss, past				
Mean ± SD	0.4 ± 0.6	0.9 ± 0.8	0.4 ± 0.7	0.087
Personal history				
Atopic diseases	27 (52.9)	5 (45.5)	10 (52.6)	0.948
AITD	15 (29.4)	2 (18.2)	6 (14.3)	0.823
Vitiligo	3 (5.9)	1 (9.1)	3 (15.8)	0.365

Abbreviations: AD, atopic dermatitis; AITD, autoimmune thyroid disease; BMI, body mass index; *N*, *n*, number of patients; SALT, Severity of Alopecia Tool.

* Statistical significance at *p* < 0.05.

** Statistical significance at *p* < 0.01.

### 3.3 Efficacy Outcomes

**FIGURE 1:**
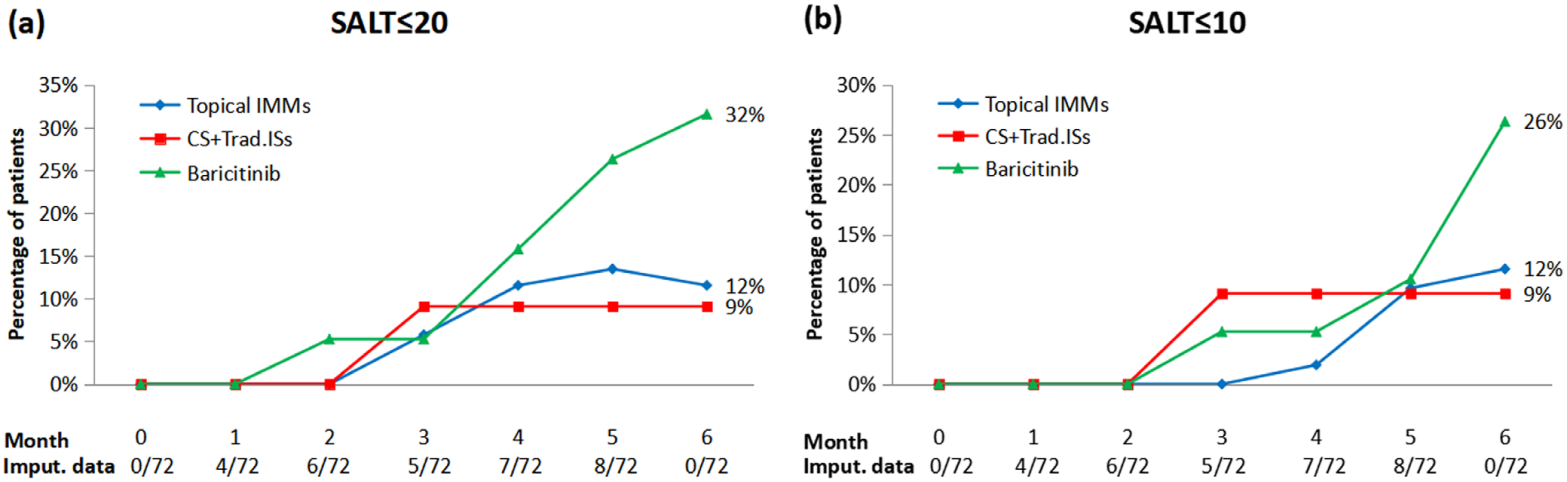
Percentage of patients achieving Severity of Alopecia Tool (SALT) score ≤ 20 (a) and SALT score ≤ 10 (b) during a 6‐month period. Number of imputed SALT scores (Imput.data) per month is also shown. Imputation techniques (individual mean substitution and last observation carried forward) were applied to missing data. CS + Trad. ISs, intramuscular corticosteroids (CS)/systemic traditional immunosuppressants (Trad. ISs); IMMs, immunomodulators.

**FIGURE 2:**
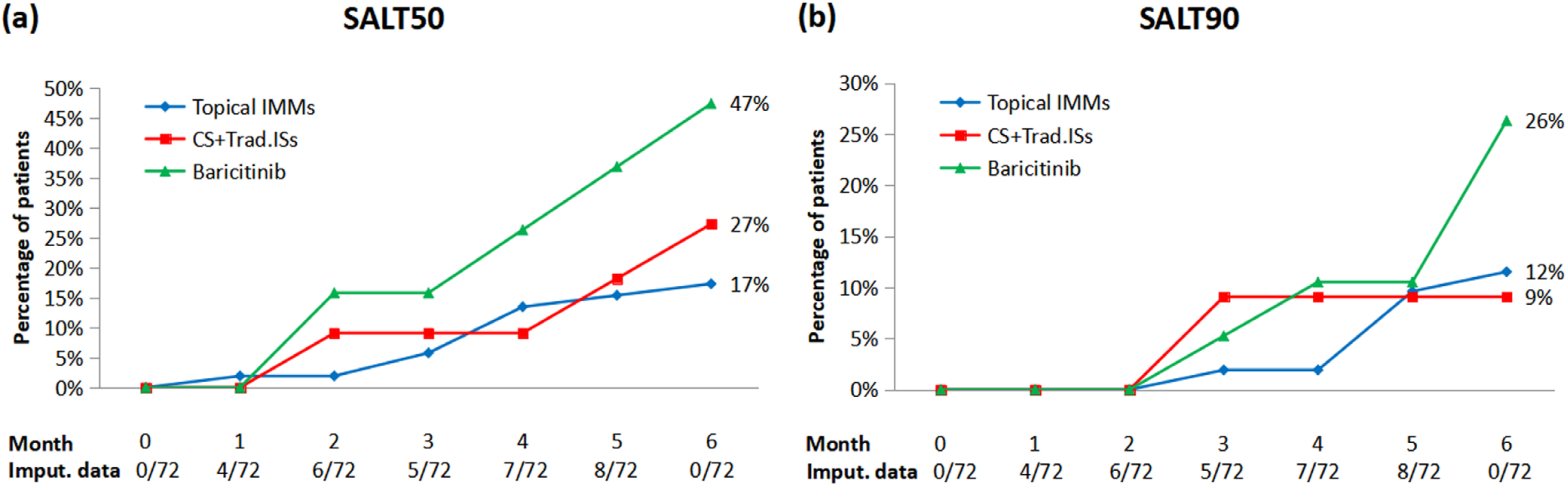
Percentage of patients achieving at least 50% (a) and 90% (b) improvement in baseline Severity of Alopecia Tool (SALT) during a 6‐month period. Number of imputed SALT scores (Imput.data) per month is also shown. Imputation techniques (individual mean substitution and last observation carried forward) were applied to missing data. CS + Trad. ISs, intramuscular corticosteroids (CS)/systemic traditional immunosuppressants (Trad. ISs); IMMs, immunomodulators.

Over the 6‐month duration, all treatment groups exhibited a progressive improvement in all efficacy endpoints. Approximately 30% of patients in the baricitinib group achieved SALT ≤ 20 and SALT ≤ 10, surpassing the rates of 12% and 9% observed in the topical IMMs and 12% and 9% in the CS + Trad. ISs groups, respectively (Figure 1a,b). In addition, a larger proportion of patients receiving baricitinib reached SALT50 and SALT90 endpoints compared to other groups. From the second month, 16% of patients in the baricitinib group achieved at least 50% improvement from baseline in contrast to 9% and 2% in CS + Trad. ISs and topical IMMs groups, respectively (Figure 2a,b). By the end of the period, almost 50% of patients in the baricitinib group met the SALT50 endpoint compared to 17% and 27% in the topical IMMs and CS + Trad. ISs groups, respectively. Similarly, percentage of patients with SALT90 after 6 months of treatment was 26% in the baricitinib group, 12% in the topical IMMs group, and 9% in the CS + Trad. ISs group.

### 3.4 Safety Profile

Treatment with topical IMMs led to transient erythema and itching in most patients (Table 4). In the DCP subgroup, these effects were observed in 37 and 40 out of 42 patients, respectively. Some patients experienced more severe reactions, with 5 reporting the presence of blisters and papules and 3 reporting headaches.

**TABLE 4:** Safety profile of treatments for severe alopecia areata over a 6‐month period.

Treatment	Adverse events (*n*)
DCP: *N* = 42	Transient erythema (37) Transient itching (40) Papules/blisters (5) Headache (3)
Baricitinib: *N* = 19	TC/LDL/TG elevation (9) URTI (4) Weight gain (3; 2 ≥ 10% body weight, 1 < 10% body weight. All within first 2 months) Acne (2) UTI (1)
DCP + anthralin cream: *N* = 9	Itching (3)
Methylprednisolone acetate: *N* = 5	None reported
Clobetasol foam: *N* = 4	Headache (1)
Methotrexate: *N* = 4	GI disturbances (1) Mild ALT/AST elevation (1) Leucocytopenia (1)
Cyclosporine: *N* = 1	None reported
Azathioprine: *N* = 1	None reported
Minoxidil solution: *N* = 1	Scalp irritation (1)
Vitamin D cream: *N* = 1	None reported
Anthralin cream: *N* = 1	None reported

Abbreviations: ALT, alanine aminotransferase; AST, aspartate aminotransferase; DCP, diphencyprone; GI, gastrointestinal; LDL, low‐density lipoprotein; *N*, number of patients; TC, total cholesterol; TG, triglycerides; URTI, upper respiratory tract infections; UTI, urinary tract infections.

In the baricitinib group, nearly 50% of patients experienced serum lipid elevations, while 25% reported short‐lasting infections, including upper respiratory tract infections (URTI) or urinary tract infections (UTI). A subset of patients reported weight gain (3/19) and acne (2/19) over the treatment period.

Elevations in liver transaminases, leucocytopenia, and gastrointestinal (GI) disturbances were observed in 25% of patients using methotrexate.

## 4 Discussion

Our data show that baricitinib treatment for severe AA outperformed both CS + Trad. ISs and topical IMMs in all efficacy outcomes by the end of the 6‐month period. All treatments were generally well‐tolerated, with mild adverse reactions not requiring permanent drug withdrawal. Patients receiving baricitinib had to take transient pauses from treatment (less than a week) during URTI/UTI episodes, while a methotrexate‐treated patient required dose adjustments after developing leucocytopenia.

The efficacy of baricitinib was demonstrated in two randomized, double‐blinded, placebo‐controlled, Phase 3 trials involving 1200 patients with severe AA (BRAVE‐AA trials) [11]. Almost 30% (28.4%) of patients who received baricitinib 4 mg daily achieved SALT ≤ 20 at Week 24. The results are comparable to those with our study at 6 months (32%). On the contrary, the percentage of patients with SALT ≤ 10 at Month 6 in our study was higher than in the BRAVE‐AA trials (26% vs. 19.0%, respectively). Differences were also found in SALT50 and SALT90 efficacy outcomes. In the BRAVE‐AA studies, more than 22% of patients experienced at least 50% improvement in hair loss at Week 12 and approximately 24% achieved at least 90% improvement by Week 36. In contrast, our study reported a 15% achievement in SALT50 at Month 3 and a 47% attainment in SALT90 at Month 6. These differences may be influenced by variations in baseline characteristics and sample sizes between the two studies.

In terms of safety of baricitinib treatment, our patients experienced serum lipid elevations (nine patients), mild‐to‐moderate URTI/URI infections (five patients), weight gain (three patients), and acne (two patients). All of these side effects are referred to the summary of product characteristics document of baricitinib but weight gain was not observed during BRAVE‐AA studies on severe AA [11, 12]. Leptin, an important regulator of body weight, acts via the JAK2–STAT3 pathway [13]. In addition, interleukin 6, a pro‐inflammatory cachectogenic cytokine, acts through JAK2 receptors [14, 15]. JAKi effectively treated cancer‐associated anorexia and adipose wasting in mice by targeting Interleukin 6 and leptin metabolism [16]. Selective JAK3 inhibition, unlike JAK2, did not lead to weight gain in humans [17]. In a recent report, gain of ≤ 10% body weight was observed in six out of seven rheumatoid arthritis patients treated with baricitinib but not with tofacitinib, a JAK1/3 inhibitor [18]. Finally, mood changes because of hair growth or loss could impact serotonin levels which could lead to weight gain [19]. The prevalence of weight gain in AA patients receiving baricitinib should be further investigated with Phase 4 studies.

Immunotherapy with topical IMMs, such as DCP has been traditionally used in patients mainly with severe AA but their reported efficacy varies between studies. In a prospective study, 3 out of 22 patients (13.6%) with severe AA achieved SALT50 after 6 months of weekly application of DCP, which is slightly lower than the outcome of our study (17%) [20]. Another study reported 22.5% of clinically significant hair regrowth at 6 months of DCP therapy but it included patients with all forms of AA [21]. Higher efficacy was obtained by another DCP study, with 39.5% of patients with severe AA achieving significant hair growth (SALT ≤ 20) after 12 months [22]. More successful outcomes were reported by Abd El‐Magid, Mohamed, and Elsharkawy [23], who reported 54% achievement of SALT ≤ 25 after 6 months of DCP treatment in patients with severe AA.

The combination of DCP with anthralin may lead to better outcomes. In a recent randomized trial, 43 patients with moderate‐to‐severe AA applied either DCP or DCP with anthralin for 6 months [24]. By the end of the study period, 47% of patient under combination treatment achieved more than 50% hair regrowth (SALT50), compared to 31% of those applying only DCP. These results are significantly higher than in our study (17%) but cannot be directly comparable as their study also included 16% of patients with moderate hair loss (24 < SALT < 50). Conversely, in another trial with 12‐ to 24‐week duration, 88% of patients on DCP achieved SALT50 in contrast to 64% of patients being on DCP‐anthralin combination [25]. These results highlight the diversity of responses in efficacy of topical IMMs in AA.

Other traditional treatment options for AA are CS and Trad. ISs. CS can be given as topical formulations, intralesional injections or systemically in different doses either continuously or as pulses. They can be safe and very effective in severe AA [26, 27]. Before the approval of baricitinib, continuous oral administration of prednisolone was a first‐choice treatment in patients with severe AA with a high relapse rate after withdrawal [28]. Patients in most studies experienced transient side effects, such as dysmenorrhea, nausea, abdominal discomfort, acne, weight gain, fat redistribution, insomnia, weakness, and arthralgia [26]. Treatment with pulsed CS could lead to less relapses and side effects than continuous administration [29]. Monthly intramuscular injections with triamcinolone acetonide 20–40 mg/mL lead to 75% hair regrowth in over 80% of 60 AA patients after 3 months [29]. Because of the absence of triamcinolone solution in our country, 5 patients received monthly intramuscular injections with methylprednisolone acetate. The diversity of treatment protocols and available CS across countries, the risk of long‐term side effects, and high percentage of disease relapses are major disadvantages preventing the wide and long‐term use of these agents [27].

Cyclosporine and methotrexate are the most preferred traditional systemic options for severe AA [28]. In a randomized double‐blinded controlled trial, 32 patients with moderate‐to‐severe AA received cyclosporine or placebo for 3 months. In the end of the study period, 31.3% of participants achieved SALT50 in contrast to 6.3% receiving placebo, which was not statistically significant [30]. According to a meta‐analysis, treatment with cyclosporine for 6 months resulted in more than 50% hair regrowth in 66% of patients but complicated by 39% relapse rate after treatment discontinuation [31]. Methotrexate can be also safe and effective option for severe AA. In a retrospective study [32], 15 patients with severe AA received methotrexate for a year. After 6 months, 5 patients achieved SALT ≤ 50 but only 1 achieved SALT ≤ 10 after 1 year. Nausea and sickness were the documented side effects in three patients but laboratory disturbances as those experienced by our patients (leukocytopenia, elevation of liver transaminases) were not mentioned. In a recent randomized controlled trial, out of 45 patients with AU/AT, only 1 achieved SALT ≤ 10 after a year with methotrexate alone in contrast to 7 out of 35 patients who received methotrexate and oral daily prednisone, without severe side effects [33].

The efficacy of traditional systemic treatments in our study was lower than most of the aforementioned studies. After 6 months, only 1 of 11 patients on either CS or Trad. ISs achieved SALT ≤ 10 (9%) and only 3 patients (27%) reported 50% improvement in hair loss (SALT50). The low number of subjects, the consistency of our group, with patients taking either CS or Trad. ISs, and the short duration of study are reasons for this difference. No studies could be also found on treating AA with intramuscular methylprednisolone injections. Also, the pulsed CS doses could be insufficient to reach a meaningful immunosuppressive threshold. However, efficacy of traditional systemic treatments is inconsistent among studies with no standard consensus on dosage and duration of treatment [34]. Combination treatment with systemic corticosteroids and Trad. ISs can be more effective but the risks of side effects after prolonged use and the high relapse rates upon withdrawal limit their use.

### 4.1 Strengths and Limitations

The primary strength of this study is that it compares the efficacy and safety of traditional treatments with baricitinib within the real‐world clinical practice of tertiary dermatological clinic, using the same data source and similar endpoints as published Phase 2/3 clinical trials, facilitating more direct comparisons. A well‐organized and designed disease‐specific registry enables the collection of valuable information on efficacy and safety, identifying potentially overlooked or underestimated side effects, such as the weight gain reported by 16% of our patients taking baricitinib.

This study has several limitations. The small size can impact the statistical power and the generalizability of our findings. While the data were extracted from a prospectively filled registry, certain sections in patient history were subject to recall bias. Approximately 5% of efficacy data had to be imputed due to missing check‐up visits. As treatment choice was non‐randomized and not blinded, our study could be subject to selection bias. As the study is observational, we can establish only association causation, for example, between weight gain and baricitinib, but cannot determine causative relationships.

## 5 Conclusion

In our retrospective cohort study, we evaluated the efficacy and safety of baricitinib, topical IMMs, CS, and Trad. ISs for severe AA. After 6 months, baricitinib was significantly superior to other options in all primary and secondary endpoints. Topical IMMs exhibited the most favorable safety profile but all treatment modalities were deemed safe, with only a minority of patients experiencing mild‐to‐moderate side effects. Continuous monitoring is needed to establish the long‐term efficacy and safety of JAKi in treating AA.

## Author Contributions

A.J.S. was involved in study conception and design, in the acquisition, analysis and interpretation of data, and drafting of the manuscript. T.D., S.G., and P.A. were involved in the critical revision of the manuscript.

## Ethics Statement

The study was reviewed and approved by the ethical review board of our hospital—University Hospital Kralovske Vinohrady (EK‐VP/40l012023), approved on August 2, 2023.

## Consent

All patients provided informed consent for the use of their data for research purposes upon their initial visit to our hospital.

## Conflicts of Interest

The authors declare no conflicts of interest.

## Data Availability

The data that support the findings of this study are available from the corresponding author upon request.
